# Large Juxtafoveal Telangiectasia

**Published:** 2010-07

**Authors:** Mohammad Hossein Jabbarpour Bonyadi, Siamak Moradian

**Affiliations:** Ophthalmic Research Center, Labbafinejad Medical Center, Shahid Beheshti University of Medical Sciences, Tehran, Iran

A 22-year-old male university student was referred due to gradual visual loss in his left eye since one year ago. His past medical and ocular history were unremarkable. On ophthalmological examination, best corrected visual acuity was 20/20 and 20/80 in the right and left eyes respectively. Anterior segment examination was unremarkable and intraocular pressure was within normal limits in both eyes.

On funduscopic examination of the left eye, diffuse parafoveal telangiectatic vessels were evident temporal to the fovea. Multiple saccular aneurysms surrounded the lesion, but no exudation was apparent (Fig. 1).

Fluorescein angiography delineated the true extent of the lesion and demonstrated some degree of leakage (Figures 2, 3). Retinal thickening temporal to the fovea was present on optical coherence tomography (OCT) (Fig. 4).

The patient was followed conservatively for a while before his vision declined to 20/160. Because of the progressive nature of the disease and decrease in visual acuity during the follow-up period, 1.25 mg bevacizumab was injected intravitreally; two weeks later, grid macular photocoagulation was applied to the area involved by telangiectasis. One month following treatment, vision was improved to 20/30 and the telangiectatic vessels regressed (Fig. 5), macular thickening resolved as documented by OCT (Fig. 6) and fluorescein angiography showed marked decrease in leakage (Figures 7, 8).

## DISCUSSION

Juxtafoveal telangiectasis (JFT) represents a group of disorders which has been classified according to ophthalmoscopic findings and natural course. Secondary causes of the condition including retinal vein occlusions, macroaneurysms, diabetic retinopathy, radiation retinopathy, intraocular inflammation, and Eales disease^1^ should be excluded to make a diagnosis of idiopathic juxtafoveal retinal telangiectasis.

Gass and Blodi^1^ proposed a classification scheme for JFT in 1993. Group 1 patients are usually white men with unilateral involvement. The area of involvement is located temporal to the fovea. The telangiectatic vessels are compromised leading to retinal exudates and edema. Loss of vision is caused by foveal involvement and macular edema, often with cystoid changes.^1^ Laser photocoagulation may help prevent accumulation of lipids in the fovea.^1^–^4^ Group 1 JFT has been suggested to be within the spectrum of Coats’ syndrome.

Group 2 JFT consists of bilateral occult telangiectasis with minimal exudation associated with small subretinal neovascular membranes.^1^ These patients are older than those in group 1 and there is no gender predilection. Visual loss results from retinal atrophy rather than exudation or retinal edema.^1^ Diabetes may be an underlying cause for group 2 idiopathic JFT.^2^,^3^

Treatment of subretinal neovascular membranes associated with idiopathic JFT using laser photocoagulation^5^ and surgery^6^ has generally been disappointing, but photodynamic therapy may be a useful treatment modality.^7^

Group 3 patients are usually female. The major finding is capillary occlusion with enlargement of the foveal avascular zone. Theoretically, photocoagulation is not beneficial in Group 3.^1^

Our patient’s visual acuity improved after treatment with intravitreal bevacizumab and grid macular photocoagulation. As mentioned earlier, in group 1 JFT, laser photocoagulation prevents the accumulation of lipids in the fovea and may stabilize vision, particularly if performed prior to accumulation of exudates in the fovea.

One important differential diagnosis is Leber’s miliary aneurysm which is an idiopathic retinal vasculopathy involving one eye of male subjects around the fourth decade of life. This condition is characterized by dilatation of venules and arterioles leading to extravasation and deposition of intraretinal exudates localized to the temporal retina between the posterior pole and the peripheral retina. In contrast to Leber’s miliary aneurysms, JFT involves the posterior pole causing earlier reduction of vision.^8^

## Figures and Tables

**Figure 1:**
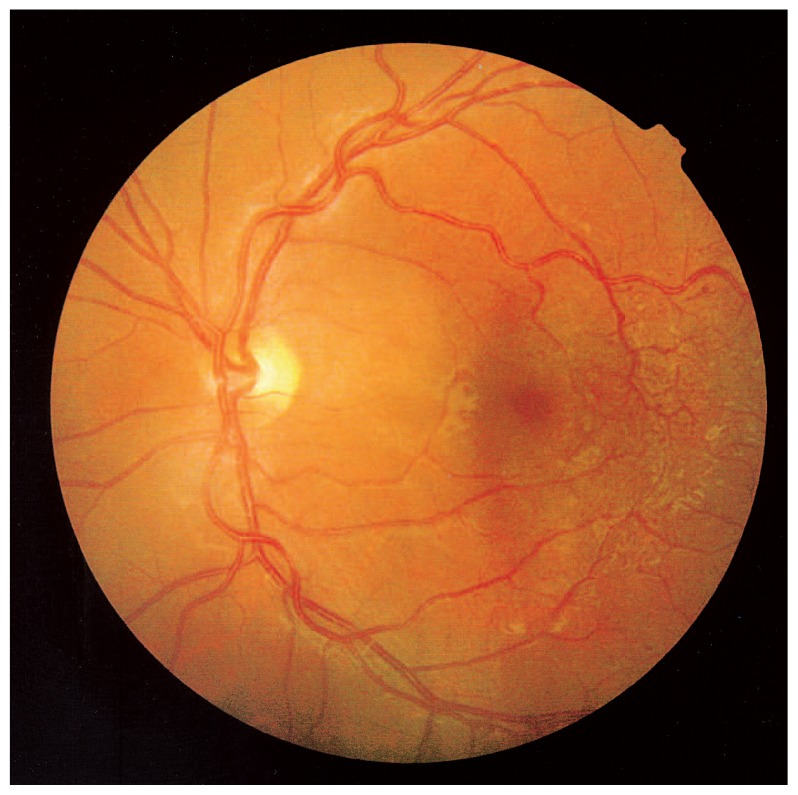
Fundus photograph showing telangiectatic vessels temporal to the macula in the left eye.

**Figure 2:**
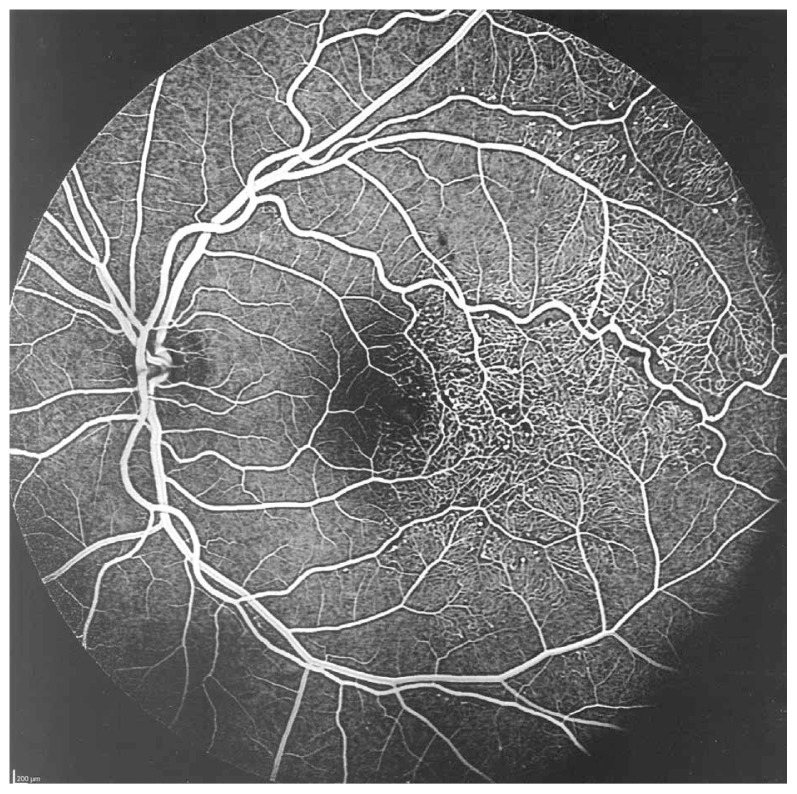
Fluorescein angiography (mid-arteriovenous phase) demonstrates extensive telangiectatic vessels.

**Figure 3:**
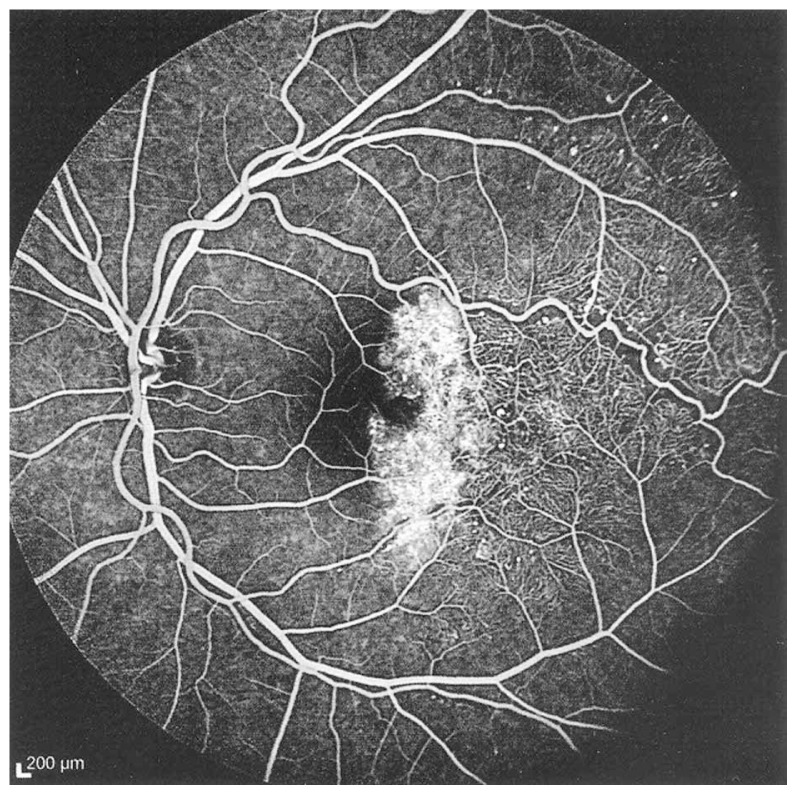
Fluorescein angiography (late venous phase) reveals leakage from the abnormal vessels.

**Figure 4:**
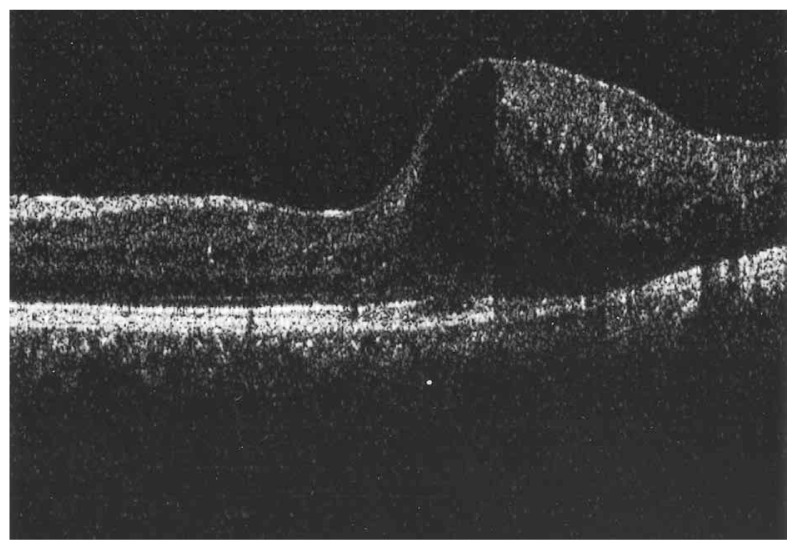
OCT of the macula, note thickening and hyperreflectivity with neurosensory detachment of the temporal macula.

**Figure 5:**
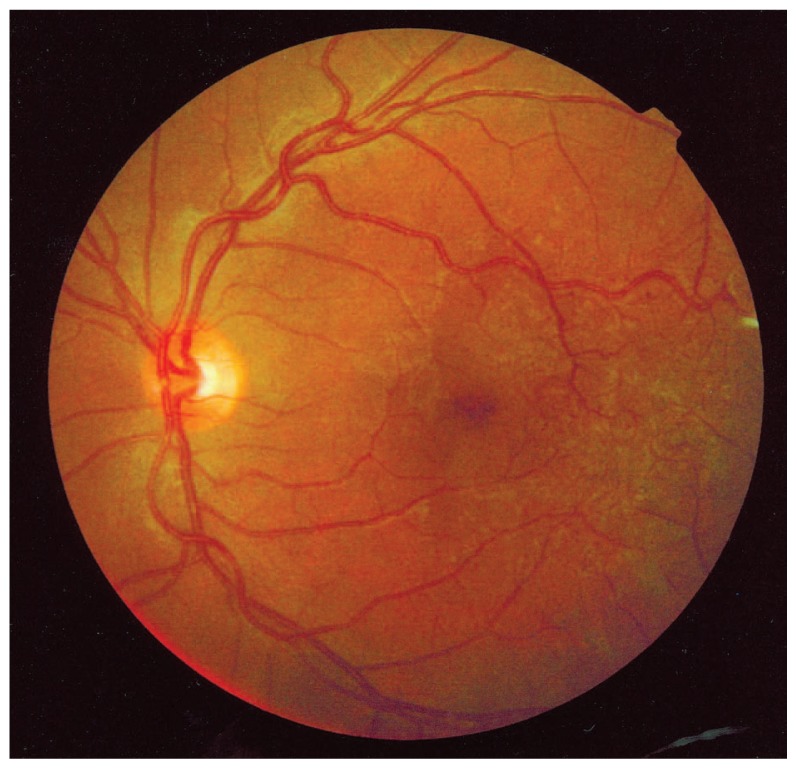
Post-treatment fundus photograph showing resolution of abnormal vessels.

**Figure 6:**
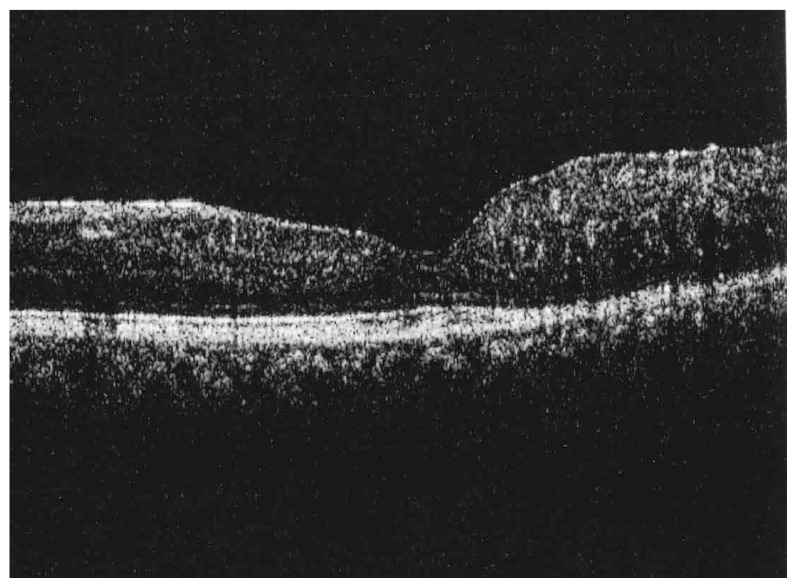
Post-treatment OCT reveals reduction in macular edema.

**Figure 7:**
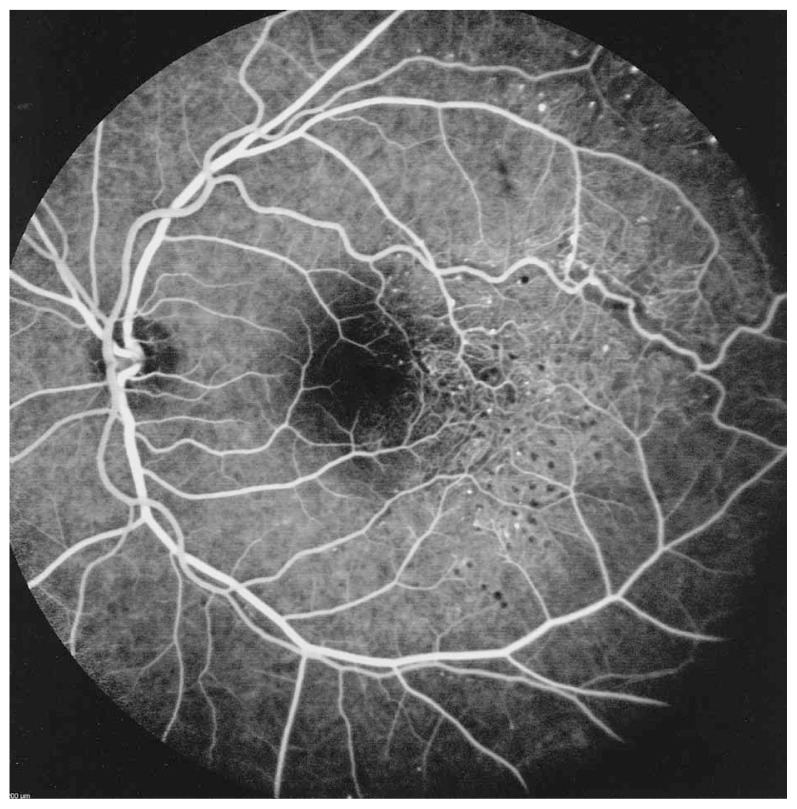
Post-treatment fundus fluorescein angiography documenting resolution of telangiectatic vessels.

**Figure 8:**
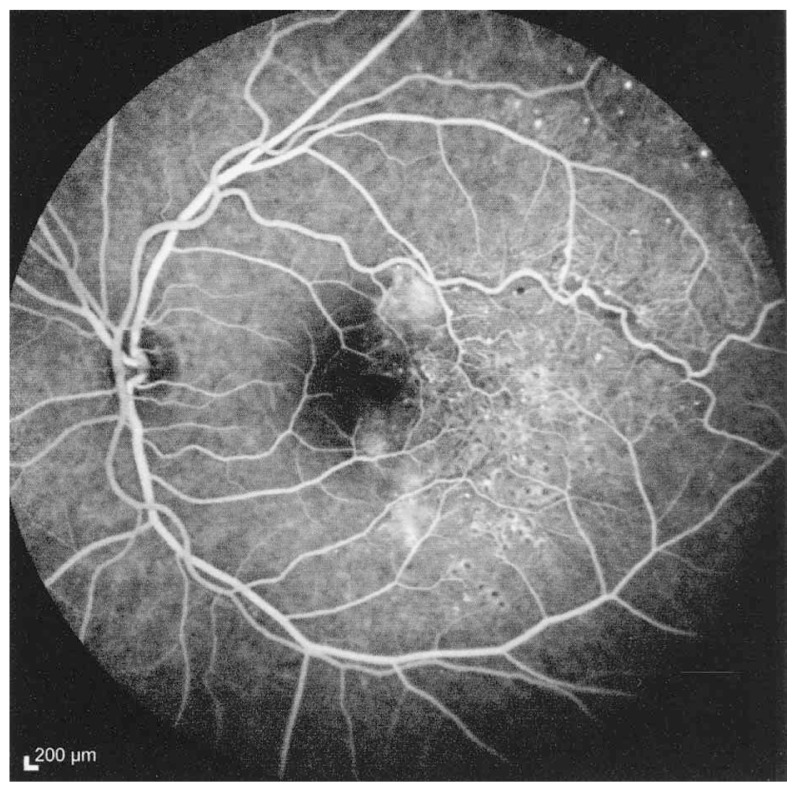
Post-treatment fluorescein angiography (late phase) demonstrates marked reduction in leakage.
